# Linking the incidence and age patterns of clinical malaria to parasite prevalence using a mathematical model

**DOI:** 10.1186/1475-2875-11-S1-P115

**Published:** 2012-10-15

**Authors:** Jamie T Griffin, Neil M Ferguson, Azra C Ghani

**Affiliations:** 1 MRC Centre for Outbreak Analysis & Modelling, Department of Infectious Disease Epidemiology, Imperial College London, UK

## Background

Estimating the changing burden of malaria disease remains difficult due to limitations in health reporting systems in those countries with the largest burden of disease. Methods extrapolating from parasite prevalence data are therefore often employed.

## Materials and methods

We present an approach to estimating disease incidence from prevalence data accounting for the changing age distribution of cases that occurs as transmission declines. We use a transmission model to capture the shifting age-pattern of disease at different transmission intensities through dynamically modelling the acquisition and loss of immunity. The model is fitted to age-stratified data on the incidence of uncomplicated malaria due to Plasmodium falciparum from 24 sites in 9 sub-Saharan African countries. We used Bayesian methods, and accounted for variation in treatment rates and reporting methods (active versus passive case detection).

## Results

We estimate that passive case detection picks up 33% (95% credible interval (Crl): 19-59%) as many cases as daily active detection, and weekly detection 76% as many (95% Crl: 61-88%). However, there was wide variation in incidence between studies that cannot be explained by differences in case-finding or case definitions such as parasitaemia thresholds, and so substantial uncertainty remains in the incidence at any given transmission intensity. We estimate that at a parasite prevalence in 2 to 10 year-olds of 60%, 55% of cases occur in under-fives and 14% in over 15s; at a prevalence of 20%, 21% are in under-fives and 41 % are in over 15s; and at a prevalence of 5%, 10% are in under-fives and 60% in over 15s.

## Conclusion

These estimates allow us to predict the incidence of clinical malaria in any age group, based on an estimate of the parasite prevalence in a possibly different age range. As the results are based on a transmission model, we can also predict the impact of interventions on incidence and its age pattern.

